# A rare case report of very low thyroglobulin and a negative whole-body scan in a patient with a solid variant of papillary thyroid carcinoma with distant metastases

**DOI:** 10.1097/MD.0000000000006086

**Published:** 2017-02-17

**Authors:** Wei Li, Danyang Sun, Hui Ming, Guizhi Zhang, Jian Tan

**Affiliations:** Department of Nuclear Medicine, Tianjin Medical University General Hospital, Tianjin, PR China.

**Keywords:** distant metastasis, solid variant of papillary thyroid carcinoma, thyroglobulin, thyroid

## Abstract

**Rationale:**

The early detection of recurrent differentiated thyroid carcinoma (DTC) cells in postsurgery DTC patients relies on the sensitivity of measuring both the level of thyroglobulin (Tg) and 131-iodine distribution on a whole-body scan (WBS). Recent studies have defined patients who subsequently have no evidence of disease as those who have a stimulated Tg level <1 ng/mL with no other radiological or clinical evidence of disease.

**Patient Concerns:**

A woman patient with solid variant papillary thyroid carcinoma (SVPTC) had undergone twice thyroidectomy with lymph node dissection and radioactive therapy. Recently, she was found to have lung and brain metastases despite a very low serum Tg level and a negative WBS. Nowadays, the patients have suggested targeted treatment, such as tyrosine kinase inhibitors, may be worthy of consideration to prevent the related events.

**Diagnoses:**

She was diagnosed as PTC.

**Interventions:**

She had undergone twice thyroidectomy with lymph node dissection and radioactive therapy.

**Outcomes:**

She was found to have lung and brain metastases despite a very low serum Tg level and a negative WBS.

**Lessons:**

We aim to suggest that patients with SVPTC should be treated cautiously because they may have a higher frequency of distant metastases and a less favorable prognosis compared with patients with classical papillary thyroid cancer.

## 1 Introduction

The most common variants of papillary thyroid carcinoma (PTC), such as classic PTC and the follicular variant, are well-differentiated tumors associated with an indolent behavior and an excellent prognosis.^[1]^ However, certain histologic variants of PTC, such as tall cell, columnar cell, and diffuse sclerosing, are considered aggressive tumors. Solid variant papillary thyroid carcinoma (SVPTC) is a rare, poorly characterized tumor that comprises approximately 3% to 13% of all PTCs.^[2,3]^ Previous studies have demonstrated the clinical utility of thyroglobulin (Tg) measurement (either TSH stimulated or nonstimulated) after total thyroidectomy (postoperative Tg) and before radioactive iodine (RAI) remnant ablation as a tool to aid in initial risk stratification and adjuvant therapy decision-making.^[4]^ The early detection of recurrent differentiated thyroid carcinoma (DTC) cells in postsurgery DTC patients relies on the sensitivity of measuring both the level of Tg and the ^131-^Iodine distribution on whole-body scan (WBS).^[5]^ We report here a patient with brain and lung metastases associated with a very low serum Tg level and a negative WBS.

### 1.1 Case

**Figure 1:**
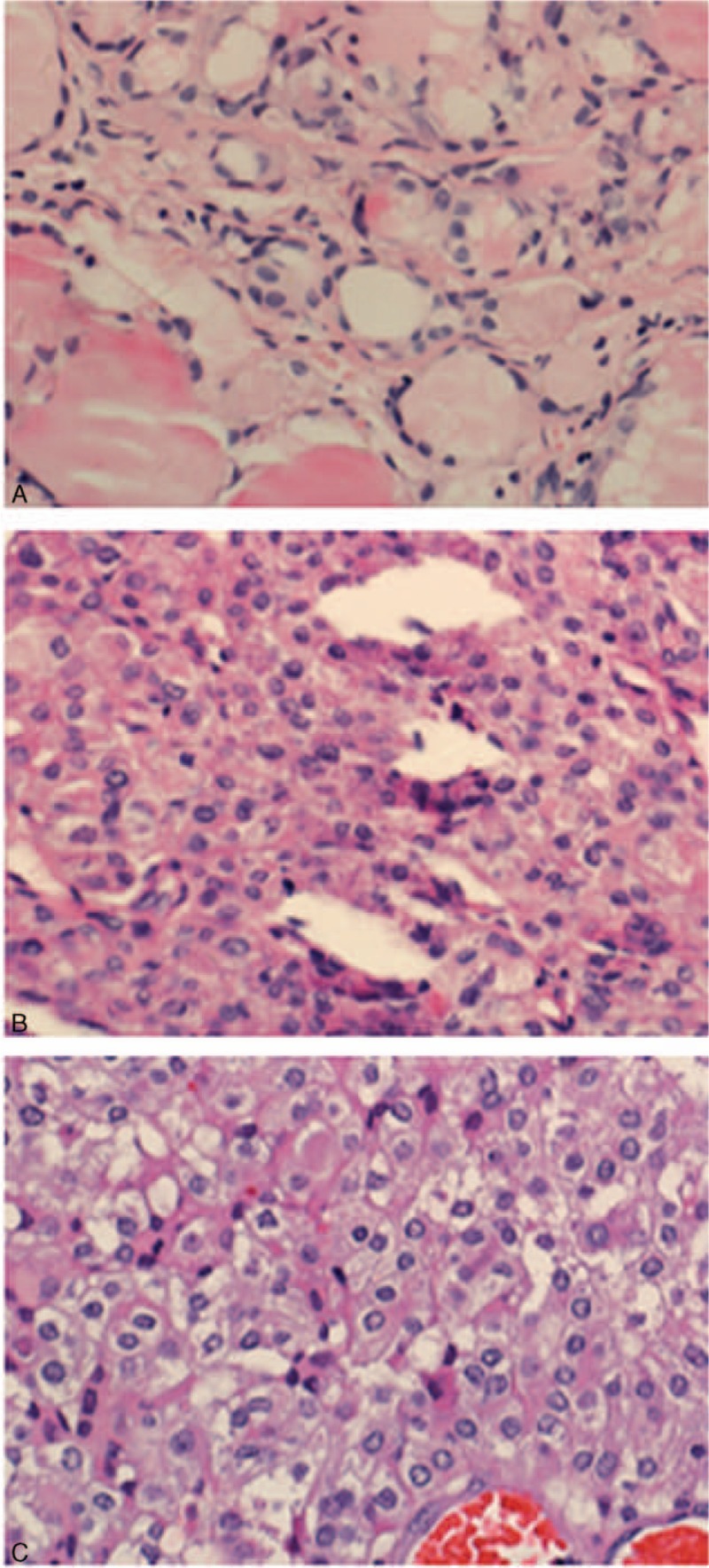
Histological variants of PTC. A, Classic PTC. Hematoxylin and eosin (H&E) stain: ×400. B, Papillary thyroid carcinoma with central lymph node metastasis and part of the tumor as solid variants. H&E stain: ×400. C, Brain lesion was PTC metastatic. H&E stain: ×400. PTC = papillary thyroid carcinoma.

A 50-year-old woman was diagnosed with PTC in the right thyroid lobe with regional lymph node metastasis and underwent right thyroidectomy and regional lymph node dissection in July of 2012. The postsurgery pathology report revealed multifocal PTCs (the maximum diameter was 0.8 cm) with extra thyroidal extension invasive and lymph node metastasis (Fig. 1A).

**Figure 2:**
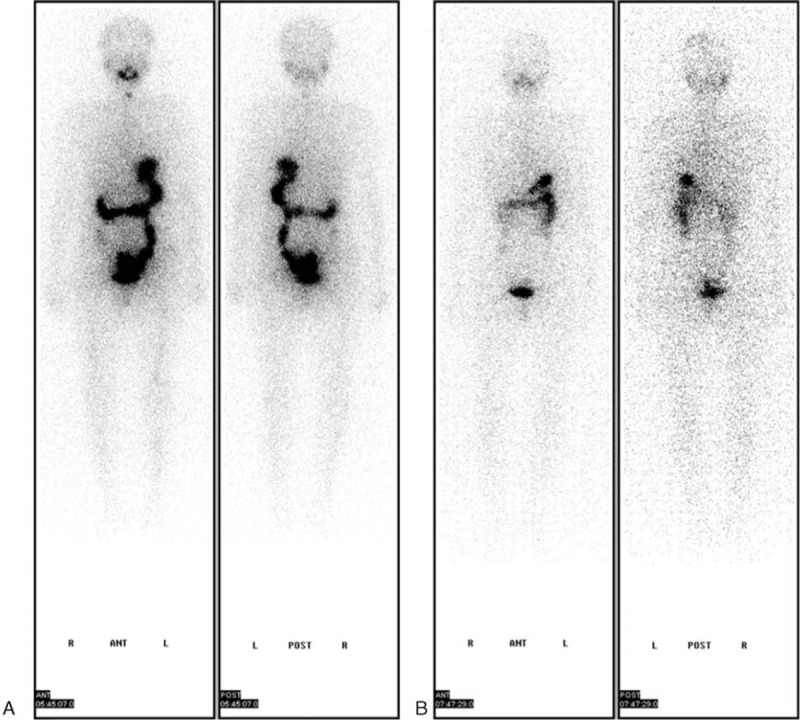
Postablation WBS after ^131^I treatment. A, WBS showed particle remnant thyroid in May of 2015, after treated with 100 mCi of ^131^1-iodine. B, WBS showed thyroid remnant activity was disappeared after 5 mCi ^131^I, and no additional sites of abnormal ^131^I uptake, especially lymph nodes, lung and brain metastases in April of 2016. WBS = 131-iodine distribution on a whole-body scan.

Three years later, she had a local recurrence in the left neck lymph nodes. Laboratory testing showed that thyroid-stimulating hormone (TSH) level was 3.99 (0.51–4.85) mIU/L, Tg level was 3.21 (1.15–130.7) ug/L, anti-Tg antibody level was <0.92 (0–4.1) IU/mL, and her calcitonin (CT) level was <2 (0–5.0) ng/L. She received left thyroidectomy plus the cervical central lymph node dissection in March of 2015. The postsurgery pathology report showed SVPTC and cervical central lymph node metastasis (Fig. 1B). Then, after discontinuation of levothyroxine for 3 weeks, laboratory testing showed that her TSH level was 136.786 (0.3–5.0) μIU/mL. Her Tg level was 0.21 (0–55) ng/mL, and her anti-Tg antibody level was <20 (0–40) IU/mL. She was treated with 100 mCi RAI in May of 2015. WBS revealed particles of remnant thyroid (Fig. 2A). Next, after her TSH suppressive therapy was restarted, laboratory testing showed that her Tg level was <0.2 ng/mL and anti-Tg antibody level was <20 IU/mL.

**Figure 3:**
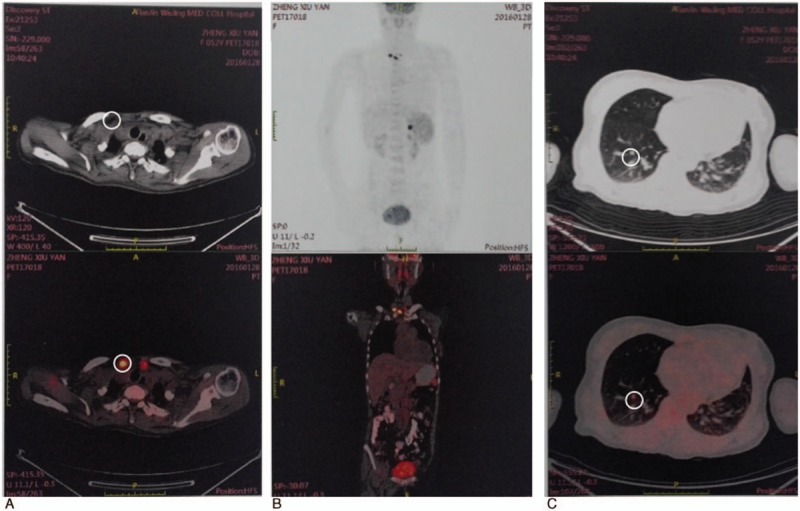
PET/CT scans. A and B, ^18^F-FDG PET/CT showed lymph metastase in the suprasternal suprasternal fossa. C, ^18^F-FDG PET/CT showed multifocal nodules in lung. PET/CT = positron emission tomography/computer tomograph.

In January of 2016, she presented with the acute onset of headache, vomiting, and paralysis on the right side. Computed tomography (CT) showed a hemorrhagic lesion in the left parietal lobe with a mass effect and surrounding oedema that extended to the anterior part of the lateral ventricles bilaterally. Immediately following, the patient underwent gross total resection of the brain lesion. The postsurgery pathology revealed a PTC brain metastasis (Fig. 1C). The tumor cells were diffusely positive for TTF-1, CK7, Tg, Ki-67, and G-CDFP. In February of 2016, ^18^F-FDG PET/CT imaging showed lymph metastasis (max SUV value was 14.0) in the suprasternal fossa and multifocal nodules in lung (the max SUV value was 2.1–2.7), and some of the nodules demonstrated abnormal FDG uptake on imaging (Fig. 3).

In April of 2016, following the discontinuation of levothyroxine for 19 days, laboratory testing showed that her TSH was 72.3 μIU/mL, her Tg level was <0.2 ng/mL, and her anti-Tg antibody level was <20 IU/mL. Two days after she took 5 mCi ^131^I, WBS revealed no additional sites of abnormal ^131^I uptake as in Fig. 2B.

The institutional review board (Tianjin Medical University General Hospital) approved this work and informed consent was given by the patient. The authors of this manuscript have no conflicts of interest.

## 2 Discussion

Tg is usually measured to evaluate the follow-up of patients with DTC. In multivariate analysis, the postoperative Tg is often found to be an independent predictor of persistent/recurrent diseases.^[4]^ High levels of postoperative stimulated Tg values (>10–30 ng/mL) are associated with poorer survival.^[6,7]^ Conversely, postoperative stimulated Tg values less than 1 to 2 ng/mL are strong predictors of remission.^[8]^ If total thyroidectomy and RAI ablation are performed, an excellent response is usually defined as a TSH-stimulated Tg level of less than 1 ng/mL in the absence of structural or functional evidence of disease (and in the absence of Tg antibodies).^[9]^ Here, we report a case of SVPTC with brain, lung, and cervical lymph node metastases associated with a very low serum Tg level and a negative WBS.

Low Tg may be noted with poorly differentiated carcinoma or nonimmunoreactive Tg. Few cases of distant metastases associated with low Tg levels have been reported in the literature, and 2 of the included reported cases had diffuse skeletal metastases.^[10,11]^ Brain metastases with very low Tg levels are uncommon, almost all of the reported brain metastases patients were noted having high Tg levels ^[8,12–14]^ It is important to underline that, in our patient, the brain metastasis was associated with a very low serum Tg level, which is the first such case to be reported.

Tg can be low in patients with metastases, Park et al^[15]^ reported a retrospective analysis of 824 consecutive patients with DTC. According to their results, only 7 of them (0.8%) had distant metastases to lung or bone with a low serum Tg level. This so-called false-negative Tg determination phenomenon can be explained by the following several reasons.^[16,17]^ First, technical issues might cause a false Tg-negative result. In particular, antigen levels 10 to 10,000 times the upper limit of the assay range can exceed the binding capacity of antibody for the solid support.^[18]^ Second, according to Brendel et al, reduced Tg synthesis and/or the release or synthesis of a Tg variant that routine radioimmunoassays cannot recognize could lead to lower Tg levels, which often occurs in marginally differentiated metastatic tumors. Alternatively, Tg might be cleared more rapidly from plasma.^[19]^ Third, the Tg structure has changed. Part of malignant transformation can change Tg's structure by reducing the iodine content and the amounts of several amino acids and monosaccharides.^[12]^ Last, small tumors are unable to secrete Tg while preserving their capability of ^131^I trapping.^[20]^ That report found that 1 g of cancer tissue elevates the serum Tg by 0.5 to 1 ng/mL, which eloquently indicates that smaller cancers secrete less Tg into the blood.^[15]^ To our patient, her tumor cells were positive for Tg on immunohistochemistry. However, the Tg level was <0.2 ng/mL, and the anti-Tg antibody level was <20 IU/mL regardless of whether TSH suppression or stimulation was administered. The low Tg level may be explained by the reduced Tg release in marginally differentiated metastatic tumors and an altered Tg structure that cannot be detected by routine methods.

Our patient is a case of SVPTC who developed a mass in the left parietal lobe that proved to be a metastasis from her PTC. Many studies have shown that SVPTC's behavior is more aggressive compared with classic PTCs.^[21]^ During the follow-up of patients with DTC,WBS and serum Tg in the hypothyroid state are performed to estimate the progression of the disease and recognize distant metastases. For our patient, it is important to emphasize that her WBS was negative and her serum Tg level was low. In such cases, a WBS or Tg level may not be helpful in establishing the clinical prognosis. The CT or ^18^F-FDG PET/CT may be more helpful in this condition.^[22]^ Brain metastasis is a rare event identified in only 0.15% to 1.3% of metastatic thyroid carcinoma patients.^[14]^ The current international guidelines (ATA 2015) recommend complete surgical resection of brain metastases as the first-line treatment.^[4]^ If surgical therapy is not feasible, alternative treatments to be discussed include radiotherapy, RAI therapy (provided a sufficient concentration of RAI) or a chemotherapy or biological therapy with tyrosine kinase inhibitors within the framework of clinical studies. In our case, the patient was amenable to surgical therapy and received 100 mCi ^131^I. Our patient with brain and lung metastases showed no additional sites of abnormal ^131^I uptake on WBS. In such cases, we suggest that alternative treatment, such as tyrosine kinase inhibitors, may be worthy of consideration to prevent the related events.

## 3 Conclusions

Patients with PTC and brain and lung metastases may also have very low serum Tg levels and negative WBS. Moreover, SVPTC patients should be treated with caution because they may have a higher frequency of distant metastases and a less favorable prognosis compared with patients with classical papillary thyroid cancer. This report suggests that neither the Tg level nor WBS can safely be used as a single marker for this PTC variant.
